# Discovering causal interactions using Bayesian network scoring and information gain

**DOI:** 10.1186/s12859-016-1084-8

**Published:** 2016-05-26

**Authors:** Zexian Zeng, Xia Jiang, Richard Neapolitan

**Affiliations:** Department of Preventive Medicine, Northwestern University Feinberg School of Medicine, Chicago, IL USA; Department of Biomedical Informatics, University of Pittsburgh, Pittsburgh, PA USA

**Keywords:** Bayesian network, Cause, Interaction, Information gain, Epistasis, Low-dimensional, Breast cancer survival

## Abstract

**Background:**

The problem of learning causal influences from data has recently attracted much attention. Standard statistical methods can have difficulty learning discrete causes, which interacting to affect a target, because the assumptions in these methods often do not model discrete causal relationships well. An important task then is to learn such interactions from data. Motivated by the problem of learning epistatic interactions from datasets developed in *genome-wide association studies* (*GWAS*), researchers conceived new methods for learning discrete interactions. However, many of these methods do not differentiate a model representing a true interaction from a model representing non-interacting causes with strong individual affects. The recent algorithm MBS-IGain addresses this difficulty by using Bayesian network learning and information gain to discover interactions from high-dimensional datasets. However, MBS-IGain requires marginal effects to detect interactions containing more than two causes. If the dataset is not high-dimensional, we can avoid this shortcoming by doing an exhaustive search.

**Results:**

We develop Exhaustive-IGain, which is like MBS-IGain but does an exhaustive search. We compare the performance of Exhaustive-IGain to MBS-IGain using low-dimensional simulated datasets based on interactions with marginal effects and ones based on interactions without marginal effects. Their performance is similar on the datasets based on marginal effects. However, Exhaustive-IGain compellingly outperforms MBS-IGain on the datasets based on 3 and 4-cause interactions without marginal effects. We apply Exhaustive-IGain to investigate how clinical variables interact to affect breast cancer survival, and obtain results that agree with judgements of a breast cancer oncologist.

**Conclusions:**

We conclude that the combined use of information gain and Bayesian network scoring enables us to discover higher order interactions with no marginal effects if we perform an exhaustive search. We further conclude that Exhaustive-IGain can be effective when applied to real data.

**Electronic supplementary material:**

The online version of this article (doi:10.1186/s12859-016-1084-8) contains supplementary material, which is available to authorized users.

## Background

The problem of learning causal influences from passive data has attracted a good deal of attention in the past 30 years, and techniques have been developed and tested. The constraint-based technique for learning Bayesian networks is a well-known method [1], and has been implemented in the Tetrad package (http://www.phil.cmu.edu/tetrad/). This method orients edges which are compelled to be causal influences. Another method for learning Bayesian networks is the *greedy equivalent search* (*GES*) [2], which does not in itself distinguish which edges are compelled to be causal. However, post-processing of its resultant network can compel edges. Both these (and other) strategies assume the *composition property*, which states that a variable *Z* and a set of variables *S* are not independent conditional on *T*, then there exists a variable *X* in *S* such that *X* and *Z* are not independent conditional on *T* [2]. When *T* is the empty set, this property simply states if *Z* and *S* are not independent then there is an *X* in *S* such that *Z* and *X* are not independent. So, at least one variable in *S* much be correlated with *Z*. However, if two or more variables interact in some way to affect *Z*, there could be little marginal effect for each variable, and the observed data could easily not satisfy the composition property. Furthermore, if interacting variables have strong marginal effects, the causal learning algorithms do not distinguish them as interactions, but only as individual causes.

So, the standard methods for learning causal influences do not learn that causes are interacting to cause a target, and do not even discover causes that are interacting with little or no marginal effect. An important task then is to learn such interactions from data. A method that does this could be a preliminary step before applying a causal learning algorithm. This paper concerns the development of a new method that does this in the case of discrete variables. We first provide some examples of situations where discrete variables interact.

### Interaction examples

An example, which has recently received a lot of attention, is gene-gene interactions, called *epistasis*. Biologically, epistasis describes a situation where a variant at one locus prevents the variant at a second locus from manifesting its effect [3]. Epistasis between *n* loci is called *pure* epistasis if none of the loci individually are predictive of phenotype and is called *strict* epistasis if no proper multi-locus subset of the loci is predictive of phenotype [4]. Epistasis has been defined statistically as a deviation from additivity in a model summarizing the relationship between multi-locus genotypes and phenotype [5]. It is believed that much of genetic risk for disease is due to epistatic interactions [6–9]. A *Single nucleotide polymorphism* (*SNP*) is a substitution of one base for another. *Genome-wide association studies* (*GWAS*) investigate many SNPs, often numbering in the millions, along with a phenotype such as disease status. By investigating single-locus associations, researchers have identified over 150 risk loci associated with 60 common diseases and traits [10–13]. However, these single-locus investigations would miss epistatic interactions with little marginal effect.

Another important example is the interaction of clinical or genomic variables with treatments to affect patient outcomes. For example, Herceptin is a treatment for breast cancer patients which is effective for HER2+ patients. So, Herceptin and HER2 status interact to affect survival. This is a well-known relationship. However, we now have large scale breast cancer and other datasets [14] from which we can learn treatment-variable interactions that are not yet known. This knowledge will enable us to better provide precision medicine.

As another example, we are now obtaining abundant hospital data concerning workflow. These data can be analysed to determine good personnel combinations and sequencing [15].

### Statistical interactions

**Fig. 1:**
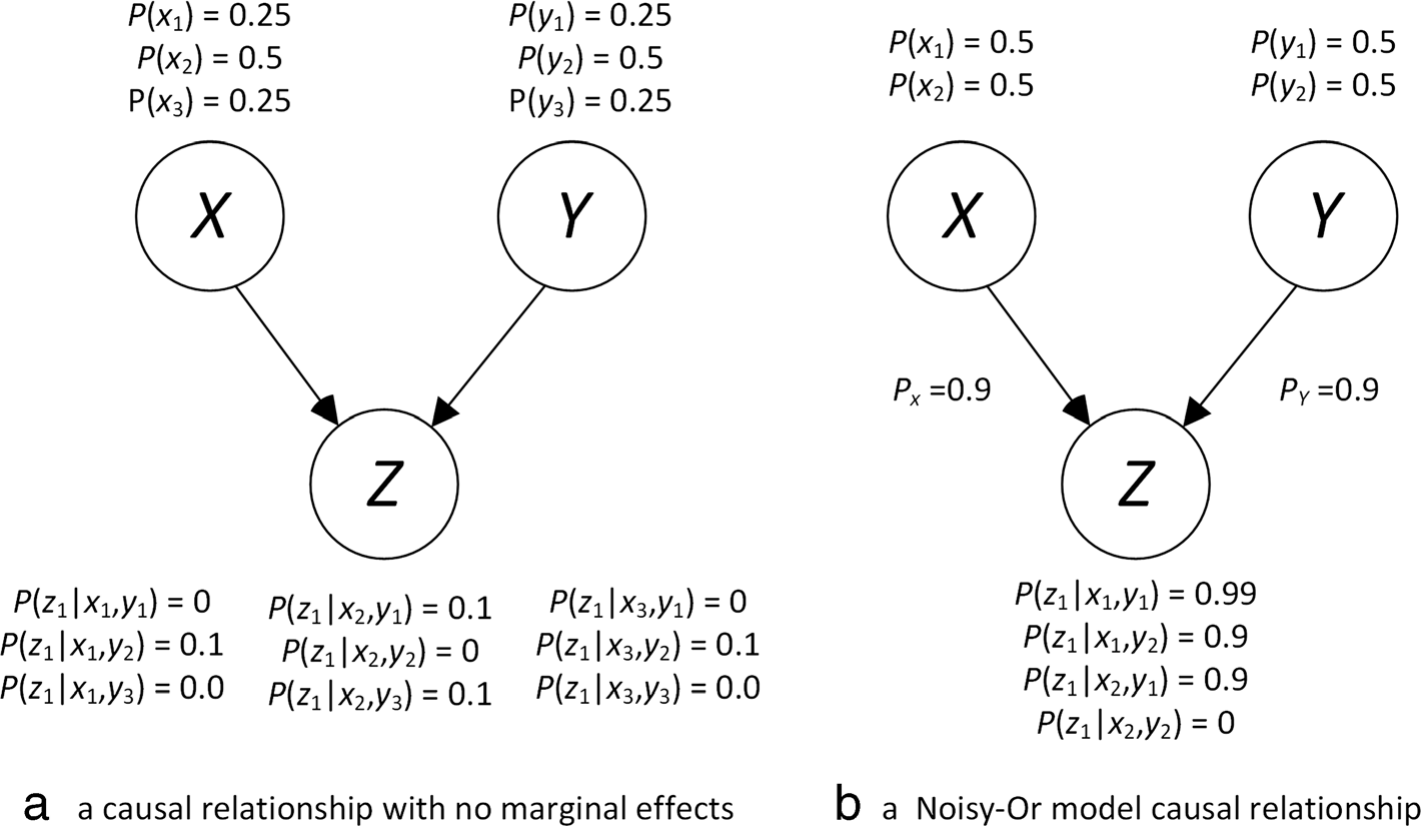
On the left is a Bayesian network representing a causal interaction with no marginal effects, and on the right is a Bayesian network representing a causal interaction described by the Noisy-Or model

In statistics, the standard definition of an interaction is a relationship where the simultaneous influence of two or more variables on a target variable is not additive. However, when we leave the domain of regression and deal with the type of non-linear discrete interactions discussed above, this definition is limited. For example, researchers have developed the Noisy-Or model to combine the effect of binary causes that are independently causing a binary target [16]. We would not call this relationship an interaction; yet the rule for combining the individual effects is not additive. When variables combine to affect a target with no marginal effect (e.g. pure, strict epistasis), we definitely can say there is an interaction. Figure 1 shows Bayesian networks illustrating these two disparate situations. We discuss Bayesian networks further in the Methods Section. However, briefly a Bayesian networks consists of nodes which represent random variables, edges between the nodes, and the conditional probability distribution of every variable given each combination of values of its parents. Figure 1a shows a causal relationship with no marginal effects. That is,

$$ P\left({z}_1\Big|{x}_1\right)=0\times 0.25+0.1\times 0.5+0\times 0.25=0.05 $$

$$ P\left({z}_1\Big|{x}_2\right)=0.1\times 0.25+0.0\times 0.5+0.1\times 0.25=0.05 $$

$$ P\left({z}_1\Big|{x}_3\right)=0.0\times 0.25+0.1\times 0.5+0.0\times 0.25=0.05. $$

By the symmetry of the problem, we see the same result holds for *Y*. Figure 1b shows a causal relationship developed with the Noisy-Or model. That model assumes each cause has a causal strength that independently affects the target. See [16] for the details of the assumptions. In this case the causal strength of *X* is *p*_*x*_ = 0.9 and the causal strength of Y is *p*_*y*_ = 0.9. From these causal strengths, the Noisy-Or model computes the conditional probabilities of *Z* as follows:

$$ P\left({z}_1\Big|{x}_1,{y}_1\right)=1-\left(1-0.9\right)\left(1-0.9\right)=0.99 $$

$$ P\left({z}_1\Big|{x}_1,{y}_2\right)=1-\left(1-0.9\right)=0.9 $$

$$ P\left({z}_1\Big|{x}_2,{y}_1\right)=1-\left(1-0.9\right)=0.9 $$

$$ P\left({z}_1\Big|{x}_2,{y}_2\right)=1-1=0 $$

The examples just shown are two extreme cases, providing us with clear examples of an interaction and a non-interaction. However, in general, there does not appear to be a dichotomous way to classify a discrete causal relationship as an interaction or a non-interaction. So, we propose a fuzzy set membership definition of a discrete interaction in the Methods Section.

### Previous research on learning discrete interactions

The problem concerning learning genetic epistasis from GWAS datasets has recently inspired ample research on learning discrete interactions from high-dimensional datasets. Researchers applied standard statistical techniques including logistic regression [17,18], and regularized logistic regression [19,20]. However, many felt that regression may not work well at learning interacting loci because the assumptions in these models are too restrictive. So researchers applied machine learning strategies including modeling full interactions [21], using information gain [22], a technique called SNP Harvester [23], using ReliefF [24], applying random forests [25], a strategy called predictive rule inference [26], a method called *Bayesian epistasis association mapping* (*BEAM*) [27], the use of maximum entropy [28], Bayesian network learning [29–31], and Bayesian network learning combined with information gain [32]. A well-known new technique called *Multifactor Dimensionality Reduction* (*MDR*) [33] was also developed. MDR combines two or more variables into a single variable (hence leading to dimensionality reduction); this changes the representation space of the data and facilitates the detection of nonlinear interactions among the variables. MDR has been applied to detect epistatically interacting loci in hypertension [34], sporadic breast cancer [35], and type II diabetes [36]. Jiang et al. [37] evaluated the performance of 22 Bayesian network scoring criteria and MDR when learning two interacting SNPs with no marginal effects. Using 28,000 simulated datasets and a real Alzheimer's GWAS dataset, they found that several of the Bayesian network scoring criteria performed substantially better than other scores and MDR. The BN score that performed best was the Bayesian Dirichlet equivalence uniform score, which is based on the probability of the data given the model.

Henceforth, we refer to a candidate cause as a *predictor*. The *multiple beam search algorithm* (*MBS*) was developed in [29] to discover causal interactions. MBS starts by narrowing down the number of predictors using a Bayesian network scoring criterion (discussed in the Methods Section) to identify a best set of possible predictors. Next it starts a beam from each of these predictors. It performs greedy forward search on this beam by adding the predictor that increases the score the most. It stops when no predictor addition increases the score. Next MBS does greedy backward search on each beam by deleting the predictor that increases the score the most. It stops when no predictor deletion increases the score. The set of predictors discovered in this manner is a candidate causal interaction. However, if two predictors each have a strong individual effect, they will have a high score together and will therefore be identified as an interaction, even if they do not interact. MBS-IGain [32] resolves this difficulty. MBS-IGain also used MBS to develop beams and uses Bayesian network scoring to end the forward search. However, it uses information gain to choose the next predictor rather than adding the predictor that increases the score the most. In a comparison using 100 simulated 1000-predictor datasets with 15 interacting predictors involved in 5 interactions, MBS-IGain substantially outperformed nine epistasis learning methods including MBS [29], LEAP [31], logistic regression [18], MDR [33] combined with a heuristic search, full interaction modeling [21], information gain alone [22], SNP Harvester [23], BEAM [27], and a technique that uses maximum entropy [28].

## Methods

MBS-IGain requires some marginal effect to detect interactions containing more than two predictors. If the dataset is not high-dimensional, we can alleviate this difficulty by instead doing an exhaustive search while using the model selection criteria in MBS-IGain. However, the exhaustive search is not straightforward because we must not only score each candidate model *M*, but also check the submodels of *M* to see how much information is provided if we do not combine them into *M*. We develop Exhaustive-IGain, which does this.

We compare the performance of Exhaustive-IGain to MBS-IGain using 10 simulated 40-predictor datasets based on 5 interactions with marginal effects, 16 simulated 40-predictor datasets based on two predictors interacting with no marginal effects, 16 simulated 40-predictor datasets based on 3 predictors interacting with no marginal effects, and 16 simulated 40-predictor datasets based on 4 predictors interacting with no marginal effects. We use Exhaustive-IGain to learn interactions from a real clinical breast cancer dataset.

Since Exhaustive-Gain uses Bayesian networks and information gain, we first review these.

### Bayesian networks

**Fig. 2:**
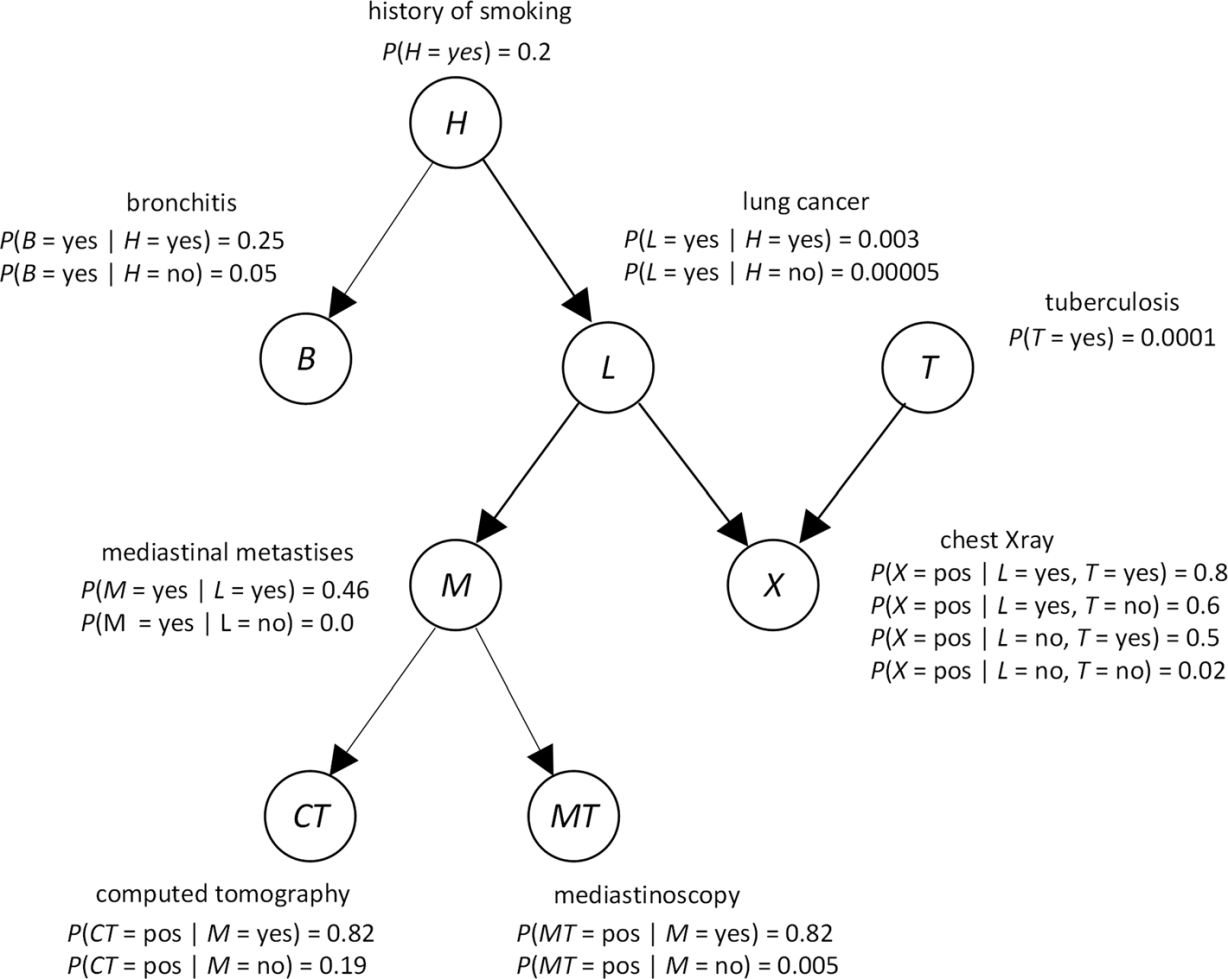
A Bayesian network representing the relationships among a small subset of variables related to respiratory illnesses

Bayesian networks [16,38–40] are an important architecture for reasoning under uncertainty in machine learning. They have been applied to many domains including biomedical informatics [41–46]. A *Bayesian network* (*BN*) represents a joint probability distribution by a *directed acyclic graph* (*DAG*) *G* = (*V*,*E*), where the nodes in *V* are random variables and the edges in *E* represent relationships among the variables, and by the conditional probability distribution of every node *X* ∈ *V* given every combination of values of the node’s parents. The edges in the DAG often represent causal relationship [16]. A BN modeling causal relationship among variables related to respiratory diseases appears in Fig. 2.

Using a BN, we can determine probabilities of interest with a BN inference algorithm [16]. For example, using the BN in Fig. 1, if a patient has a smoking history (*H* = yes), a positive chest X-ray (*X* = pos), and a positive CAT scan (*CT* = pos), we can determine the probability of the patient having lung cancer (*L* = yes). That is, we can compute *P*(*L* = yes| *H* = Yes, *X* = pos, *CT* = pos). Inference in BNs is NP-hard [47]. So, approximation algorithms are often employed [16].

Learning a BN from data concerns learning both the parameters and the structure (called a DAG model). In the score-based structure-learning approach, a score is assigned to a DAG based on how well DAG model *G* fits the *Data*. The Bayesian score [48] is the probability of the *Data* given *G*. This score, which uses a Dirichlet distribution to represent prior belief concerning each conditional probability distribution in the BN, follows:

$$ scor{e}_{Bayes}\left(G\kern1em :\kern1em  Data\right)=P\left( Data\Big|G\right)={\displaystyle \prod_{i=1}^n}{\displaystyle \prod_{j=1}^{q_i}}\frac{\varGamma \left({\displaystyle {}_{k=1}^{r_i}}{a}_{ijk}\right)}{\varGamma \left({\displaystyle {}_{k=1}^{r_i}}{a}_{ijk}+{\displaystyle {}_{k=1}^{r_i}}{s}_{ijk}\right)}{\displaystyle \prod_{k=1}^{r_i}}\frac{\varGamma \left({a}_{ijk}+{s}_{ijk}\right)}{\varGamma \left({a}_{ijk}\right)} $$

where *n* is the number of variables in the model, *r*_*i*_ is the number of states of *X*_*i*_, *q*_*i*_ is the number of different values that the parents of *X*_*i*_ can jointly assume, *a*_*ijk*_ is a hyperparameter, and *s*_*ijk*_ is the number of times *X*_*i*_ assumed its *k* th value when the parents of *X*_*i*_ assumed their *j* th value. When *a*_*ijk*_ = *α*/*r*_*i*_*q*_*i*_, where *α* represents a prior equivalent sample size, we call the Bayesian score the *Bayesian Dirichlet equivalent uniform* (*BDeu*) score [49].

It has been shown that the problem of learning a BN DAG model from data is NP-hard [50]. Resultantly, heuristic search algorithms have been developed [16].

### Information gain, interaction strength, and interaction power

Information theory [51] concerns the quantification and communication of information. Given a discrete random variable *Z* with *m* alternatives, the *entropy H*(*Z*) is defined as follows:

$$ H(Z)=-{\displaystyle \sum_{i=1}^mP\left({z}_i\right){ \log}_2P\left({z}_i\right).} $$

If we repeat *n* trials of the experiment having outcome *Z*, then it is possible to show that the entropy *H*(*Z*) is the limit as *n* → ∞ of the expected value of the number of bits needed to report the outcome of every trial. Entropy provides a measure of our uncertainty in the value of *Z* in the sense that, as entropy increases, it takes more bits on the average to resolve our uncertainty. Entropy achieves its maximum value when *P*(*z*_*i*_) = 1/*m* for all *z*_*i*_, and its minimum value (0) when *P*(*z*_*j*_) = 1 for some *z*_*j*_.

The expected value of the entropy of *Z* given *X* is called the conditional entropy of *Z* given *X*. We denote conditional entropy as *H*(*Z* | *X*). Mathematically, we have

$$ H\left(Z\Big|X\right)={\displaystyle \sum_{j=1}^kH\left(Z\Big|{x}_j\right)}P\left({x}_j\right), $$

where *X* has *k* alternatives. Knowledge of the value of *X* can reduce our uncertainty in *Z*. The *information gain* of *Z* relative to *X* is defined to be the expected reduction in the entropy of *Z* given *X*:

$$ IG\left(Z;X\right)=H(Z)-H\left(Z\Big|X\right). $$

Let *IG*(*Z*;*X*,*Y*) denote the information gain of *Z* relative to the joint probability distribution of *X* and *Y*. The *interaction strength* (*IS*) of *X* and *Y* relative to *Z* as then defined as follows:

$$ IS\left(Z;X,Y\right)=IG\left(Z;X,Y\right)-IG\left(Z;X\right)-IG\left(Z;Y\right). $$

Let *IG*(*Z*;*A*) denote the information gain of *Z* relative to the joint distribution of all variables in set *A*. The *IS* of variable *X* and set of variables *A* is then defined as follows:

$$ IS\left(Z;X,A\right)=IG\left(Z;X,A\right)-IG\left(Z;X\right)-IG\left(Z;A\right). $$

Since *A* is a set, *A* ∪ {*X*} should technically be used in the *IG* expression. However, we represent this union by *X*, *A*. Interaction strength provides a measure of the increase in information gain obtained when *X* and *A* are known together relative to knowing each of them separately.

When *IG*(*Z*;*M*) ≠ 0, we define the *interaction power* (*IP*) of model *M* for effect Z as follows:

$$ IP\left(Z;M\right)=\underset{A\subset M}{ \min}\frac{IS\left(Z;M-A,A\right)}{IG\left(Z;M\right)}=\underset{A\subset M}{ \min}\frac{IG\left(Z;M\right)-IG\left(Z;M-A\right)-IG\left(Z;A\right)}{IG\left(Z;M\right)}. $$

Since information gain (*IG*) is nonnegative, it is straightforward that *IP*(*Z*;*M*) ≤ 1. If *M* is causing *Z* with no marginal effects (e.g. pure, strict epistasis), the *IP* is 1. We would consider this a very strong interaction. When the *IP* is small, the increase in *IG* obtained by considering the variables together is small compared to considering them separately. We would consider this a weak interaction or no interaction at all.

Jiang et al. [32] show that if the variables in *M* are independent causes of *Z*, then

$$ IS\left(Z;M-A,A\right)\ge 0. $$

So, in situations we often investigate, the *IP* is between 0 and 1, and therefore satisfies the notion of a fuzzy set [52], where the greater the value of the *IP* the greater membership the model has in the fuzzy set of interactions.

The *IS* and *IP* can be used to discover interactions. In this next section we develop algorithms for learning interactions that use the *IS* and the *IP*.

### Interaction strength algorithms

We present the *multiple beam search information gain* (*MBS-IGain*) and *exhaustive search information gain* (*Exhaustive-IGain*) algorithms, which use information gain and Bayesian network scoring to learn interactions. MBS-IGain, which was previously developed in [32], does a heuristic search, while Exhaustive-IGain does an exhaustive search.

**Fig. 3:**
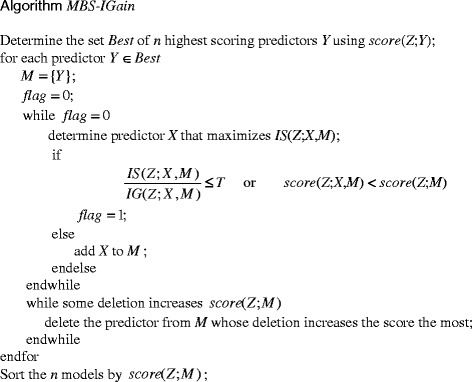
Algorithm MBS-IGain

Figure 3 shows Algorithm MBS-IGain. The *score*(Z;*M*) in Algorithm MBS-IGain is the BDeu score of the DAG model that has the predictors in *M* being parents of the target *Z*. The notation *score*(*Z*:*Y*) indicates that *Y* is the only parent of *Z*. MBS-IGain symbiotically uses the *IS* and *IG* functions and a Bayesian network scoring criterion. Initially, the most promising predictors are chosen using the scoring criterion. A beam is then started from each of these predictors. On each beam, the predictor, which has the highest *IS* with the set of predictors chosen so far, is greedily chosen. The search ends when either the *IS* is small relative to the *IG* of the model (based on a threshold *T*), indicating that the *IP* would be small, or when adding the predictor decreases the score of the model. This latter criterion is included because we not only want to discover predictors that seem to be interacting, but we also want to discover probable models. On the other hand, the check for a sufficiently large *IS* is performed because a set of SNPs could score very high as parents of *Z* when there is no interaction. For example, if *X* and *Y* each have strong causal strengths for *Z* but affect *Z* independently, the model with them as parents of *Z* would score high. The Noisy-OR model [16] is such a model. In this situation the model *X* → *Z* ← *Y* would have a high score without there being an interaction. Finally, a parameter *R*, which puts a limit on the size of the model *M* learned, could be included in MBS-IGain.

**Fig. 4:**
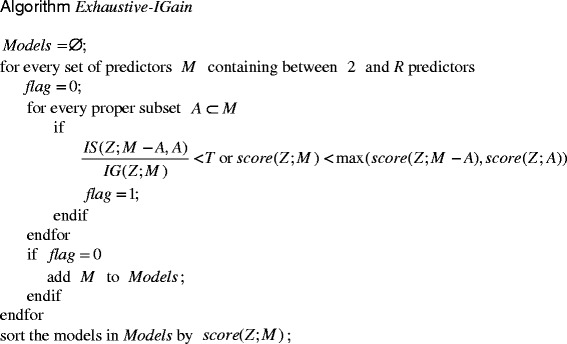
Algorithm Exhaustive-IGain

MBS-IGain will miss a 3-predictor or 4-predictor pure epistatic interaction. When there are not many predictors, we can ameliorate this problem by doing an exhaustive search. Algorithm Exhaustive-IGain, which appears in Fig. 4, does this. The parameter *R* is the maximum size of the interactions we are considering. For each set *M* of size between 2 and *R*, the algorithm checks every subset *A* of *M* to see if the ratio of *IS*(*Z*;*M* ˗ *A*,*A*) to *IG*(*Z*;*M*) exceeds a threshold *T*. In this way it makes certain that the *IP* exceeds *T*. It also checks that *M* yields a higher score than both *A* and *M*-*A*. If *M* passes these tests for every subset, then *M* is considered an interaction.

### Reporting the noteworthiness of an interaction

**Fig. 5:**
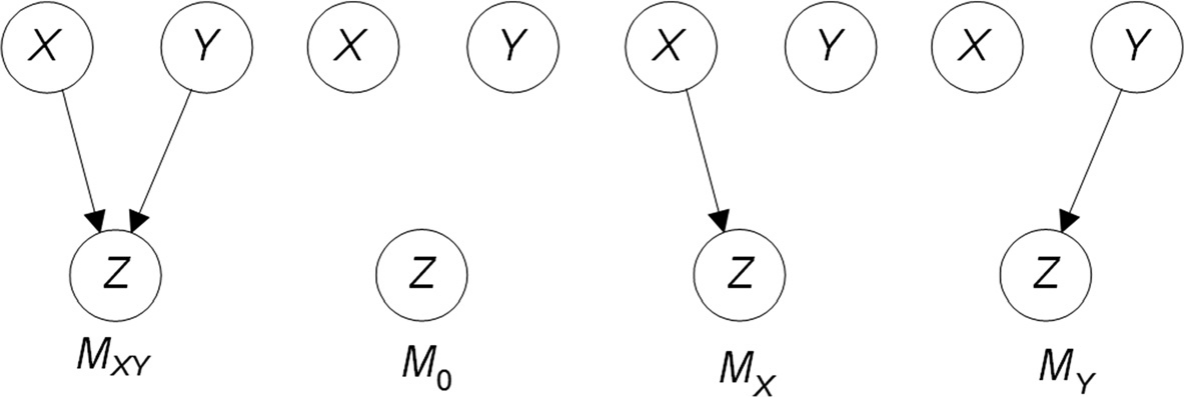
The model that *X* and *Y* are both parents of *Z* is on the left, and its three competing models are on the right

Once we discover an interaction, we need to report its noteworthiness. First, we report its *IP* to indicate its strength as an interaction. However, if the model is unlikely, it is still not very noteworthy even if the *IP* is large. So, we also need to in some way report the significance of the model. Standard *p*-values are not very informative because there is more than one null hypothesis. Consider Fig. 5, which shows the DAG model *M*_*XY*_ in which *X* and *Y* are both parents of *Z*. The three competing models are on the right. Model *M*_0_ represents that neither variable is a parent of *Z*, Model *M*_*X*_ has *X* as a parent of *Z* and *Y* not as a parent of *Z*, and model *M*_*Y*_ has *Y* as a parent of *Z* and *X* not as a parent of Z. Standard statistical techniques do not investigate these multiple competing hypotheses. They only pit the null hypothesis *M*_0_ against *M*_*XY*_. However, if either model *M*_*X*_ or *M*_*Y*_ were the correct model, we would obtain an association of the two variables together with *Z* (and thus reject *M*_0_) even though *M*_*XY*_ is incorrect. Towards addressing this difficulty, Jiang et al. [30] developed the *Bayesian network posterior probability* (*BNPP*), which provides the posterior probability of a DAG model that has an arbitrary number of parents of a target *Z*. For the two-parent model *M*_*XY*_, the BNPP is as follows:

$$ P\left({M}_{XY}\Big| Data\right)=\frac{P\left( Data\Big|{M}_{XY}\right)P\left({M}_{XY}\right)}{P\left( Data\Big|{M}_{XY}\right)P\left({M}_{XY}\right)+P\left( Data\Big|{M}_0\right)P\left({M}_0\right)+{\displaystyle {\sum}_kP\left( Data\Big|{M}_k\right)P\left({M}_k\right)}}, $$

where *k* sums over the two 1-predictor models. The BNPP extends to larger models, but the number of competing hypotheses grows exponentially with size of the model. However, in general, we usually don’t learn an interaction with more than 5 predictors. Jiang et al. [30] discuss and provide prior probabilities in the case of interactions learned from GWAS datasets.

### Evaluation methodology

We evaluated Exhaustive-IGain by comparing it to MBS-IGain using simulated datasets, and by applying it to a real breast cancer dataset. We discuss each of these next.

### Simulated datasets

One hundred simulated datasets based on interacting trinary variables causing a binary target were developed by Chen et al. [53]. They labeled the predictors SNPs and the target a disease. Therefore, we will proceed with this terminology. Each dataset had 1000 total SNPs, and consisted of 1000 cases and 1000 controls. The datasets were generated based on two 2-SNP interactions, two 3-SNP interactions, and one 5-SNP interaction, making a total of 15 causative SNPs. The effects of the interactions were combined using the Noisy-Or model [16]. The 5 interactions used to generate the datasets were as follows:

1. S1, S2, S3, S4, S5
2. S6, S7, S8
3. S9, S10, S11
4. S12, S13
5. S14, S15

Each of these 5 interactions exhibits some marginal effect. As mentioned in the Introduction Section, MBS-IGain [33] previously outperformed 9 other methods at interaction discovery using these 100 datasets. We developed 10 datasets based on these same interactions, but with only 40 total SNPs. Each dataset has 1000 cases and 1000 controls.

Urbanowicz et al. [54] created GAMETES, which is a software package for generating pure, strict epistatic models with random architectures. We used GAMETES to develop 2-SNP, 3-SNP, and 4-SNP models of pure epistatic interaction. That is, there are no marginal effects. The software allows the user to specify the *heritability* and the *minor allele frequency* (*MAF*). We used values of heritability ranging between 0.01 and 0.2, and values of MAF ranging between 0.1 and 0.4. Using these values, we generated 16 datasets based on pure, strict 2-SNP interactions, 16 datasets based on pure, strict 3-SNP interactions, and 16 datasets based on pure, strict 4-SNP interactions. The 2-SNP and 3-SNP based datasets contained 1000 cases and 1000 controls, and the 4-SNP based datasets contained 5000 cases and 5000 controls. All the simulated datasets are available in Additional file 1.

We used both MBS-IGain and Exhaustive-IGain to analyze both sets of datasets. We ran both algorithms with all combination of the following values of the threshold *T* in the algorithms: *T* = 0.1, 0.2; and the parameter α in the BDeu score: α = 9, 54, 128.

We compared the results using the following two performance criteria:

- **Criterion 1:** This criterion determines how well the method discovers the predictors in the interactions, but does not concern itself with whether the method discovers the actual interactions. First, the learned interactions are ordered by their scores. Then each predictor is ordered according to the first interaction in which it appears. Finally, the power according to criterion 1 is computed as follows:
  $$ Powe{r}_1(K)=\frac{1}{H\times M}{\displaystyle \sum_{i=1}^H{N}_K(i)} $$
  where *N*_*K*_(*i*) is the number of true interacting predictors appearing in the first *K* predictors learned for the *i*th dataset, *M* is the total number of interacting predictors in all interactions, and *H* is the number of datasets.
- **Criterion 2:** This criterion measures how well a method discovers each of the interactions. The criterion used the Jaccard index which is as follows:
  $$ Jaccard\left(A,B\right)=\frac{\#\left(A\cap B\right)}{\#\left(A\cup B\right)}. $$

The Jaccard index equals 1 if the two sets are identical and equals 0 if their intersection is empty. The criterion provides a separate measure for each true interaction. The learned interactions are first ordered by their scores for each dataset *i*. Denote the *j*th learned interaction in the *i*th dataset by *M*_*j*_ (*i*), and denote the true interaction we are investigating by C. For each *i* and *j* we compute *Jaccard*(*M*_*j*_(*i*),*C*). We then set

$$ {J}_K\left(i,C\right)=\underset{1\le j\le K}{ \max}\kern0.5em  Jaccard\left({M}_j(i),C\right). $$

The power according to criterion 2 for interaction *C* is then computed as follows:

$$ Powe{r}_2\left(K,C\right)=\frac{1}{H\times M}{\displaystyle \sum_{i=1}^H{J}_K\left(i,C\right)} $$

where *H* is the number of datasets and *M* is the total number of interacting predictors in interaction *C.*

### Real dataset

The METABRIC data set [15] has clinical data and outcomes for 1981 primary breast cancer tumors. Table 1 shows the clinical variables and their values used in our analysis. The data in three of these variables were transformed from their original METABRIC values using domain knowledge and the equal distribution discretization strategy. The transformations follow:

- *age_at_diagnosis*: This variable was discretized to the five ranges shown using the equal distribution discretization technique and breast cancer expert knowledge.
- *size*: This variable was discretized to the three standard ranges shown.
- *lymph_nodes_positive*: This variable was grouped into the six ranges shown.

**Table 1:** The clinical variables in the METABRIC dataset

Variable	Description	Values
age_at_diagnosis	age at diagnosis of the disease	0-39, 39–54, 54–69, 69–84, 84-100
menopausal_status	inferred menopausal status	pre, post
size	size of tumor in cm	0-20, 20–50, 50-180
lymph_nodes_positive	number of positive lymph nodes	0, 1, 2–3, 4–5, 6–9. ≥ 10
lymph_nodes_removed	number of lymph nodes removed	0, 1–3, 4–9, 10–20, ≥ 21
percent_nodes_positive	percent of removed nodes positive	0-0.2, 0.2-0.4, 0.4-0.6, 0.6-0.8, 0.8-1
grade	grade of disease	1, 2, 3
stage	composite of size and # positive nodes	0,1,2,3,4
histological	tumor histology	IDC, Other
ER_Expr	estrogen receptor expression	+, −
PR_Expr	progesterone receptor expression	+, −
HER2_status	HER2 expression	+, −
P53_mutation_status	whether P53 is mutated	+, −
chemo	whether patient had chemotherapy	yes, no
radiation	whether patient had radiation therapy	yes, no
hormone	whether patient had hormone therapy	yes, no

The outcome variable is whether the patient died from breast cancer. If the person was known to die from breast cancer, the days after initial consultation that the patient died is recorded. If the person was not known to die from breast cancer, the days after initial consultation that the patient was last seen alive or died from another cause is recorded. If a patient was known to die from breast cancer within *x* years after initial consultation or is known to be alive *x* years after initial consultation, we say their breast cancer survival status is known *x* years after initial consultation. These data provide us with 1698 patients whose breast cancer survival status is known 5 years after initial consultation, 1228 patients whose breast cancer survival status is known 10 years after initial consultation, and 782 patients whose breast cancer survival status is known 15 years after initial consultation.

We used Exhaustive-IGain to learn interactions that affect 5 year, 10 year, and 15 year breast cancer survival.

## Results and discussion

### *S*imulated datasets based on marginal effects

**Fig. 6:**
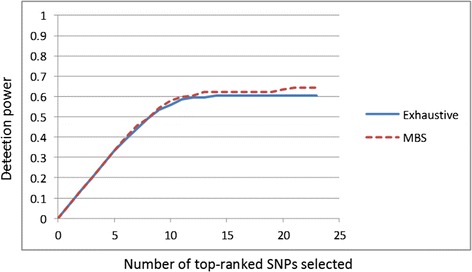
Comparison of Exhaustive-IGain and MBS-IGain, when analysing the simulated datasets based on interactions with marginal effects, using Performance Criterion 1

**Fig. 7:**
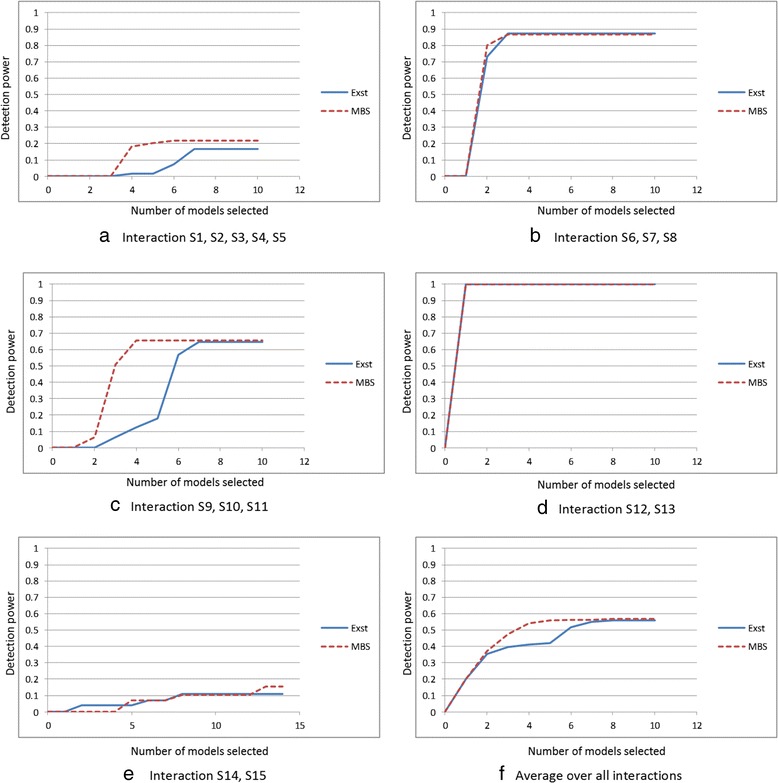
Comparison of Exhaustive-IGain and MBS-IGain, when analysing the simulated datasets based on interactions with marginal effects, using Performance Criterion 2

The results were similar for all combinations of the parameters, but best when *T* = 0.2 and α = 54. Figure 6 shows *Power*_1_(*K*) for *K* ≤ 25 for the Exhaustive-IGain and MBS-IGain algorithms. Figure 7 shows *Power*_2_(*K,C*) for *K* ≤ 12 for each interaction *C* for the two methods. Figure 7f shows the average of *Power*_2_(*K*,*C*) over all 5 interactions. It is initially surprising that MBS-IGain does slightly better than Exhaustive-IGain according to Power Criterion 1 and, on the average, according to Power Criterion 2. These results can be attributed to the superior performance of MBS-IGain for interaction {S1,S2,S3,S4,S5} (Fig. 7a) and interaction {S9,S10,S11} (Fig. 7c). An explanation for this superior performance is as follows. MBS-IGain, for example, could have S9 and S10 already chosen on a beam and be considering S11 next. The model {S9,S10,S11} is only checked for interaction strength relative to the models {S9,S10} and {S11}. So, if the information gain of {S9,S10,S11} satisfies a threshold relative to the sum of the information gains of {S9,S10} and {S11} (and it increases the score), the model will be chosen. On the other hand, for Exhaustive-IGain to choose model {S9,S10,S11}, that model must also beat the sum of the gains for {S9,S11} and {S10} and the sum of the gains for {S10,S11} and {S9}. So, MBS-IGain will more readily accept model {S9,S10,S11}. This is a property of these particular datasets, and should not indicate the MBS-IGain performs better than Exhaustive-IGain when there are marginal effects. MBS-IGain does a heuristic search, and the performance of a comparison to each combination of sub-models, as done by Exhaustive-IGain, has a more proper theoretical basis. Note that the performances of the two methods are about the same in the case of the 2-SNP models (Fig. 7d and e), when this phenomenon cannot occur.

Exhaustive-IGain discovers on the average 7.5 models and MBS-IGain discovers on the average 7 models. When there are 40 SNPs, there about 760,058 models containing between 2 and 5 SNPs. So, both methods exhibit the good discovery performance shown in Figs. 5 and 6 with very few false positives. Note that MBS-IGain could discover at most 40 models because there are only 40 beams.

### Simulated datasets based on pure, strict epistasis

**Fig. 8:**
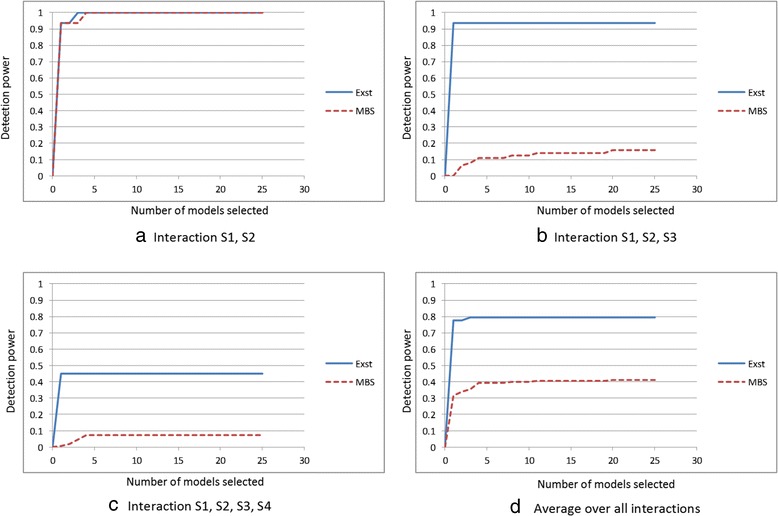
Comparison of Exhaustive-IGain and MBS-IGain, when analysing the simulated datasets based on pure epistatic interactions with no marginal effects, using Performance Criterion 2

Figure 8 shows *Power*_2_(*K,C*) for Exhaustive-IGain and MBS-IGain for *K* ≤ 25 for each of the three pure epistatic interactions.

We see from Fig. 8a that both methods discover the 2-SNP interaction very well. In fact Exhaustive-IGain ranked the correct interaction first in 15 of the datasets and 3^rd^ in the remaining dataset, while MBS-IGain ranks it first in 15 of the datasets and 4^th^ in the remaining dataset (This information is not in the figure). In the case of a 2-SNP interaction, MBS-IGain effectively does an exhaustive search, explaining why it performs almost as well as Exhaustive-IGain. Its slightly worse performance is due to its different exit criteria concerning the score. It stops adding predictors when no predictor increases the score. On the other hand, Exhaustive-IGain checks whether any sub-model has a higher score than the model being considered. Exhaustive-IGain achieves this performance with very few false discoveries. The average number of interactions discovered by Exhaustive-IGain is 2.0. On the other hand, the average number of interactions discovered by MBS-IGain is 4.75.

Figure 8b shows that Exhaustive-IGain also discovers the 3-SNP interactions extremely well, while MBS-IGain exhibits poor performance. This poor performance is to be expected. That is, when there are no marginal effects, if {S1,S2,S3} is our interaction, S2 or S3 would be chosen first on the beam initiating from S1 only by chance. In general, Exhaustive-IGain exhibited this good performance with a low false positive rate. The average number of interactions discovered for 15 of the datasets was 2.47. However, for one of the datasets, 100 interactions (the maximum reported) were identified.

As Fig. 8c shows, Exhaustive-IGain performed well for the 4-SNP interactions, but not as well it did for the smaller models. This result indicates that higher order interactions are more difficult to discover. As expected, MBS-IGain again showed very poor performance. For 14 of the datasets, the average number of interactions discovered by Exhaustive-IGain was 1.85. However, for two of the datasets, 100 interactions were discovered.

### Real dataset

Table 2 shows the correlations of each of the predictors with breast cancer survival according to both the BNPP and Pearson’s chi-square test. Except for a few exceptions, the two methods are in agreement. Our purpose here is not to discuss these correlations, but rather to provide them as a frame of reference for the learned interactions, which appear in Table 3.

**Table 2:** The individual variable effects learned from the METABRIC dataset. The p-values were obtained using the chi-square test

Variable	5 year BC death		10 year BC death		15 year BC death	
	BNPP	p-value	BNPP	p-value	BNPP	p-value
P53_mutation_status	1	0	0.97	0.001	0.936	0.0004
HER2_Status	1	0	1	0	0.853	0.0006
chemo	1	0	1	0	0.999	0
PR_category	1	0	1	0	0.971	0.002
hormone	0.880	0.112	0.410	0.120	0.999	0
radiation	0.240	0.320	0.170	1	0.280	0.576
ER_category	1	0	1	0	0.889	0.002
overall_stage	1	0	1	0	1	0
menopausal_status	0.940	0.019	0.190	0.76	0.421	0.554
histological	0.450	0.0250	0.940	0.002	0.913	0.055
lymph_nodes_pos	1	0	1	0	1	0
percent_nodes_positive	1	0	1	0	0.999	0
overall_grade	1	0	1	0	0.999	0.0001
size	1	0	1	0	0.954	0.014
age_at_diagnosis	1	0	1	0	0.950	0.0003
axillary_nodes_removed	0.160	0.113	0.950	0.003	0.147	0.567

**Table 3:** The interactions learned from the METABRIC dataset

Outcome	Interaction	BNPP	IP
5 year BC death	histological, menopausal_status	0.77	0.43
	histological, hormone	0.93	0.47
10 year BC death	hormone, menopausal_status	0.32	0.72
15 year BC death	histological, menopausal status	0.57	0.49

Table 3 shows the interactions learned from the Metabric dataset that have IPs > 0.4. The data indicates that *histological* interacts with *menopausal_status* to affect both 5 year and 15 year breast cancer death survival. A consultation with a breast cancer oncologist^1^ reveals that invasive ductal carcinoma (IDC) has a worse prognosis in premenopausal women, but other histological types do not. Furthermore, Table 2 indicates that neither *histological* nor *menopausal status* is highly correlated with 5 year or 15 year breast cancer death survival by themselves. Table 3 also shows that the data indicates *hormone* and *menopausal_status* interact to affect 10 breast cancer death survival. The breast cancer oncologist indicated that hormone therapy is more effective in post-menopausal women. As Table 2 shows, neither *hormone* nor *menopausal_status* are highly correlated with 10 year breast cancer death survival by themselves. Finally, Table 3 shows that the data indicates that *histological* and *hormone* interact to affect 5 year breast cancer death survival. The oncologist stated IDC might respond slightly worse to hormone therapy than other types, but that this difference is not well-established.

The BNPP is a relatively new concept, and the *IP* is a complete new concept. So, we do not have the same intuition for their values as we have for a p-value. That is, we have come to consider a p-value of 0.05 meaningful partly due to Fisher's [55] statement in 1921 that “it is convenient to draw the line at about the level at which we can say: Either there is something in the treatment, or a coincidence has occurred such as does not occur more than once in twenty trials,” and also due to years of experience. To provide a context for the results in Table 3, Table 4 shows the average BNPPs and IPs of all 2, 3, 4, and 5 predictor models obtained from the Metabric dataset. As we would expect, the value of the BNPP decreases as the size of the models increases. However, the IP is small for models of all sizes. The models we learned (Table 3) are all 2-predictor models. So we compare those results to the averages for 2-predictor models. Our IP results of 0.43, 0.47, 0.72 and 0.49 are all substantially larger than the 2-predictor IP average of 0.042. Three of our BNPP results, namely 0.77, 0.93, and 0.57 are much higher than the average 2-predictor BNPP of 0.266. However, the value of 0.32, which is obtained for {*hormone*, *menopausal_status*}, is not much higher than the average. Yet, this model has the largest IP (0.72).

**Table 4:** The average BNPPs and IPs of all 2, 3, 4, and 5 predictor models obtained from the Metabric dataset

Model	Avg. BNPP	Avg. IP
2-predictor models	0.266	0.042
3-predictor models	0.005	−0.005
4-predictor models	6.13 × 10 ^-7^	0.013
5-predictor models	7:04 × 10 ^-16^	0.040

## Conclusions

We compared Exhaustive-IGain to MBS-IGain using simulated datasets based on interactions with marginal effects, and simulated datasets based on interactions with no marginal effects. MBS-IGain performed as well as (actually slightly better than) Exhaustive-IGain when analysing the datasets based on interactions with marginal effects. MBS-IGain is *O*(*Rn*^2^) whereas Exhaustive-IGain is *O*(*n*^*R*^), where *n* is the number of predictors and *R* is the maximum size of the models considered. So, our results indicate that MBS-IGain achieves similar results to Exhaustive-IGain with this type of dataset, but much more efficiently. On the other hand, as could be expected, MBS-IGain could not discover pure epistatic interactions involving more than two SNPs. Exhaustive-IGain performed very well at discovering 3-SNP interactions, and reasonably well at discovering 4-SNP interactions. We conclude from these results that the combined use of information gain and Bayesian network scoring enables us to discover higher order pure epistatic interactions if we perform an exhaustive search.

When we applied Exhaustive-IGain to a real breast cancer dataset to learn interactions affecting breast cancer survival, we learned interactions that agreed with the judgements of a breast cancer oncologist. We conclude that Exhaustive-IGain can be effective when applied to real data.

## Abbreviations

BDeu, Bayesian Dirichlet equivalent uniform; BEAM, Bayesian epistasis association mapping; BN, Bayesian network; BNPP, Bayesian network posterior probability; DAG, directed acyclic graph; Exhaustive-IGAIN, exhaustive search information gain; GES, greedy equivalent search; GWAS, genome-wide association studies; IP, interaction power; IS, interaction strength; MAF, minor allele frequency; MBS, multiple beam search; MBS-IGAIN, multiple beam search information gain; MDR, Multifactor Dimensionality Reduction; SNP, single nucleotide polymorphism.

## Additional file

Additional file 1: Supplement A. (ZIP 1133 kb)

## Footnotes

1 Adam Brufsky, MD, PhD, Professor of Medicine at the University of Pittsburgh School of Medicine.
