# Supplementary material for: Discovering causal interactions using Bayesian network scoring and information gain
Source: BMC Bioinformatics. 2016 May 26;17:221. doi: 10.1186/s12859-016-1084-8 (PMC4880828; doi:10.1186/s12859-016-1084-8)
Supplement: Additional file 1: — Supplement A. (ZIP 1133 kb) [file 12859_2016_1084_MOESM1_ESM.zip › Supplement A/Test Datasets Interactions/Readme.docx]

Data Sets Base on Interactions with Marginal Effects

In each data set there are 40 SNPs, 1000 cases, and 1000 controls. Each column is a patient. The interaction models are as follows:

Model 1: S1, S2

Model 2: S3, S4

Model 3: S5, S6, S7

Model 4: S8, S9, S10

Model 5: S11, S12, S13, S14, S15

See [1] for a description of how the interactions were developed.

1. Chen et al. Comparative analysis of methods for detecting interacting loci. BMC Genomics 2011; 12:344.
