# Supplementary material for: Discovering causal interactions using Bayesian network scoring and information gain
Source: BMC Bioinformatics. 2016 May 26;17:221. doi: 10.1186/s12859-016-1084-8 (PMC4880828; doi:10.1186/s12859-016-1084-8)
Supplement: Additional file 1: — Supplement A. (ZIP 1133 kb) [file 12859_2016_1084_MOESM1_ESM.zip › Supplement A/Test Datasets Pure Epistasis/Readme.docx]

Pure Epistasis Data Sets

We have three folders:

2-SNP Models: There are 40 SNPs total, the final two are engaged in a 2-SNP pure interaction. There are 1000 cases and 1000 controls.

3-SNP Models: There are 40 SNPs total, the final three are engaged in a 3-SNP pure interaction. There are 1000 cases and 1000 controls.

4-SNP Models: There are 40 SNPs total, the final three are engaged in a 4-SNP pure interaction. There are 1000 cases and 1000 controls.
